# Accuracy of Velacur in Assessing MASLD and MASH Patients Using Biopsy as the Gold Standard

**DOI:** 10.3390/diagnostics15050615

**Published:** 2025-03-04

**Authors:** Muhammad Y. Sheikh, Nameer Hasan, Marwan Almozuaghi, Nadeem M. Akhtar, Yugjeet Grewal, Caitlin Schneider

**Affiliations:** 1 Fresno Clinical Research Center, Fresno, CA 93720, USA; liverdisease@gmail.com (M.Y.S.); nameer.hasan@gmail.com (N.H.); malmozuaghi@fresnocrc.com (M.A.); nadeema2808@gmail.com (N.M.A.); yugjeetgrewal@gmail.com (Y.G.); 2 Sonic Incytes Medical Corp., Vancouver, BC V6J 1P2, Canada

**Keywords:** MASLD, MASH, metabolic syndrome, non-invasive tests, liver stiffness, quantitative ultrasound

## Abstract

**Background/Objectives**: Velacur^TM^ is a novel, point-of-care ultrasound device developed to accurately diagnose patients with Metabolic Dysfunction-Associated Steatotic Liver Disease (MASLD) and Metabolic Dysfunction-Associated Steatohepatitis (MASH). The Velacur system non-invasively assesses liver stiffness, attenuation, and the Velacurdetermined fat fraction (VDFF). In this study, the performance of Velacur was measured against biopsy results in a cohort of MASLD and MASH patients. **Methods**: This prospective study enrolled adult patients who were scheduled to undergo biopsy within 6 months of enrollment. The primary objective was to validate Velacur’s findings against that of histological findings. The secondary objective was to compare Velacur results with those of FibroScan. **Results**: A total of 78 participants were enrolled, and 70 were included in the analysis. Patients had a mean age of 53.3 ± 13.1 years, with a mean BMI of 35.0 ± 6.24 kg/m^2^. A total of 11, 19, 13, 25, and 2 were characterized as F0 to F4, respectively. The mean Velacur stiffness was 6.48 ± 1.4 kPa, and the mean VDFF was 14.4 ± 5.1%. In patients with significant fibrosis the Velacur AUC [95% CI] was 0.86 [0.76, 0.93] and 0.79 [0.66, 0.88] for patients with advanced fibrosis. For measurements of steatosis, 2, 24, 20, and 24 patients were found to have S0 to S3, respectively. To determine moderate steatosis (≥S2), the VDFF had an AUC of 0.846 [0.716, 0.920]. In the comparison population (n = 59), VDFF (0.85 [0.72, 0.94]) was significantly different than FibroScan CAP (0.50 [0.35, 0.66]) for the detection of moderate steatosis. **Conclusions**: This study validates the use of Velacur as a non-invasive tool for assessment of steatosis and fibrosis, hallmarks of MASLD and MASH, when compared to histological evidence provided via hepatic biopsy. Further, Velacur outperformed FibroScan in the assessment of steatosis.

## 1. Introduction

Metabolic Dysfunction-Associated Steatotic Liver Disease (MASLD), previously known as non-alcoholic fatty liver disease, encompasses a spectrum of conditions. This range generally begins with steatosis, defined by ≥5% hepatic fat content along with comorbidities, and progresses to Metabolic Dysfunction-Associated Steatohepatitis (MASH), which involves hepatic inflammation [1,2]. Over time, patients can develop fibrosis and advance to cirrhosis, which can eventually lead to liver failure and/or cancer [1,2]. MASLD is the fastest growing cause of chronic liver disease in the world, affecting approximately 25–30% of the global population [3]. The condition is particularly prevalent among individuals with metabolic syndrome, type 2 diabetes mellitus, obesity, dyslipidemia, and/or hypertension [4,5,6].

In 2023, the American Association for the Study of Liver Diseases established guidelines for the non-invasive evaluation of patients at risk for MASLD or those incidentally diagnosed with liver steatosis in the absence of other chronic liver diseases [4,7]. Primary risk assessments, such as the FIB-4 score, and secondary assessments, including the Enhanced Liver Fibrosis test [8] and Vibration-controlled Transient Elastography (VCTE), primarily used with FibroScan [9], assist in stratifying clinically significant fibrosis. Additional risk evaluation may involve imaging studies such as MRI-Proton density fat fraction (MRI-PDFF) [10], Magnetic Resonance Elastography (MRE) [11,12,13], and multi-parametric MRI [14]. However, these MRI based studies are expensive and not readily available in a clinical point of care setting.

Emerging data and guidelines increasingly support the use of Non-invasive Tests (NITs) to identify the severity of fibrosis, the key prognostic feature in patients with MASH [15,16]. Liver stiffness is strongly correlated with liver fibrosis development and is commonly used in clinical practice to estimate liver fibrosis staging [17]. Additionally, ultrasound attenuation serves as a surrogate measure for assessing liver fat content [18,19].

Despite the trend in using NITs, MASH can only be diagnosed histologically, and the FDA still considers liver biopsy as the gold standard in assessing the endpoint in clinical trials [20]. So, although ultrasound elastography and ultrasound attenuation measurements have been proven to be an effective clinical point of care solution in estimating liver disease, it is still important to validate new measures against the biopsy gold standard.

Several types of ultrasound-based liver assessment have emerged as valuable clinical tools. Among these include acoustic radiation force impulse (ARFI) imaging and 2D shear wave imaging, which both use acoustic force to produce shear waves within the liver [17]. These methods are often available on diagnostic ultrasound imaging machines [21]. Shear wave elastography has been well characterized and is part of current guidance for MASH and MASLD assessment [4]. In a point of care environment, FibroScan (EchoSens, Paris, France) is widely known and often used as a screening tool for clinical trials. Several new devices are being developed and marketed; among these is Velacur (Sonic Incytes Medical Corp., Vancouver, BC, Canada). Velacur is a new commercially available, point-of-care, ultrasound-based elastography assessment tool designed for evaluating patients with chronic liver disease. A recent study compared the performance of Velacur and FibroScan using MRE and MRI-PDFF as the ground truth. The study found that Velacur outperformed FibroScan in detecting steatosis and demonstrated equivalent efficacy in identifying advanced fibrosis [22]. Velacur has also recently introduced a new measure: Velacur determined fat fraction (VDFF) [23]. This new measurement was also used in the study presented here to assess patient liver fat when compared to biopsy.

This is the first study to validate the performance of Velacur using recent liver biopsies in a cohort of patients with MASLD or MASH.

## 2. Materials and Methods

### 2.1. Study Design

This prospective open label study recruited consecutive patients at Fresno Clinical Research Center (Fresno, CA, USA). Initially, patients with suspected or confirmed MASLD/MASH who had or were planning to undergo a biopsy within 3 months were enrolled. Later the time frame for biopsy was widened to within 6 months. Patients received a Velacur scan during their clinical appointment before or after the liver biopsy.

The primary objective of this study was to validate the performance of Velacur elasticity, attenuation, and VDFF measurements against the results of liver histology. The secondary objective was to compare the performance of Velacur to FibroScan.

This study was conducted in accordance with Good Clinical Practices. Informed consent, in writing, was obtained from each patient, and the study protocol conformed to the ethical guidelines of the Declarations of Helsinki and Istanbul. This study was approved a priori by an appropriate institutional review committee (WCG #1333729, 23 May 2022).

### 2.2. Patient Selection

Adults between the ages of 18 and 75, of any sex, were enrolled. Patients must have some evidence of MASLD or chronic liver disease, including any of the following: (1) previous biopsy proven disease, (2) evidence of hepatic steatosis from abdominal imaging, FibroScan (CAP > 230 dB/m) or MRI-PDFF (>12%) measurements, or (3) at least 2 criteria for metabolic syndrome and increased stiffness on FibroScan (>8 kPa), within 12 months. Metabolic syndrome criteria include BMI ≥ 25 kg/m^2^ (or the ethnicity-adjusted equivalent), Type 2 diabetes, blood pressure ≥ 130/85 (or hypertensive treatment), plasma triglycerides ≥ 1.70 mmol/L (or lipid-lowering treatment), plasma HDL-cholesterol ≤ 1.0 mmol/L (M), and ≤1.3 mmol/L (F) (or lipid-lowering treatment) [24].

All patients needed to have had, or be scheduled for, a biopsy within 6 months of the Velacur scan. The biopsy was being performed for an alternative reason and not performed directly as part of this study.

All participants must have been able to understand the informed consent form and the study procedures and must have been willing to participate in this study.

Exclusion criteria included current significant alcohol consumption. Patients with a history of significant alcohol consumption for a period of more than 3 consecutive months at any time within 1 year prior to enrollment were excluded. For this study, significant alcohol consumption was defined as more than 20 g per day in females and more than 30 g per day in males, on average [4]. Alcohol consumption was assessed by the physician, patient medical records, and patient interviews. Other exclusion criteria included a history of cirrhosis and clinical evidence of hepatic decompensation or acute hepatitis. Patients using drugs historically associated with MASLD were also excluded.

### 2.3. Velacur Imaging

Velacur is a commercially available ultrasound-based tool intended for the assessment of liver tissue properties. Velacur is able to measure liver tissue stiffness, ultrasound attenuation, and VDFF. Shear Wave Absolute Vibro-Elastography (S-WAVE) is the ultrasound elastography method used to measure liver tissue stiffness. S-WAVE creates a multi-frequency steady state shear wave using a vibration source placed under the patient. Using a sweep motion in the elevational direction of the probe, Velacur captures a large volume of the right lobe of the liver. From the ultrasound data, quantitative parameters can be measured, such as attenuation and backscatter. In addition to attenuation alone, VDFF uses a combination of attenuation and backscatter measurements, collected simultaneously, to estimate the liver fat content.

Throughout this study, Velacur scans were performed by trained personnel of the Fresno Clinical Research Center. Velacur operators were trained by a certified Sonic Incytes trainer. Operators were instructed to collect 10 volumes (each volume is used to create a single elasticity, attenuation, and VDFF measure). Within the software of the device, an objective machine-learning-based algorithm was used to assess the overall quality of the collected volume. This algorithm was able to measure the shear wave quality in the volume and a goodness-of-fit measure for attenuation and VDFF [25]. A predetermined threshold, calibrated during the creation of the shear wave algorithm, was used to determine if the scan was considered to be valid. The final result presented in this paper was the median value of all the collected volumes that were above the quality threshold. Quality of the elasticity and ultrasound measures can be assessed separately. At least 3 of the 10 total collected volumes needed to be above the threshold for the patient to have a valid measurement and be included in the analysis.

### 2.4. Biopsy

Biopsies were performed percutaneously under either ultrasound or CT guidance. As biopsies were not performed specifically for this study, biopsies for this study were interpreted by several different pathology labs and pathologists. All biopsies were deemed to be of sufficient quality to complete the assessment. The NASH CRN scoring system was used in all cases to quantify the results for steatosis, lobular inflammation, and ballooning [26,27]. Only grading for steatosis is presented here, and steatosis was graded on a scale of 0–3 based on the percentage of hepatocytes showing steatosis. Fibrosis was staged from F0 to F4 based on the location of fibrosis present as well as the fibrosis architecture [27].

A minimum of 6 pathologists from 2 laboratories were involved in the readings. The reports were gathered from the patient’s records to be used for this study. If a report designated a patient as 1a, 1b, or 1c, they were included into the F1 group for the purpose of analysis.

Many biopsies were performed for screening or endpoints of other trials. A single, local pathology laboratory was used, when possible, to improve interpretation consistency.

### 2.5. Statistical Methods

The analysis population is made up of patients with both biopsy results (within 183 days) and a valid Velacur scan. The percentage of invalid scans and patients who were enrolled but did not complete a biopsy within the required time frame, is reported. An invalid Velacur scan was defined as an exam in which at least 3 of the collected volumes did not pass the pre-defined quality threshold as defined by the objective machine-learning-based shear wave detection algorithm [25].

For each stage of fibrosis, according to the biopsy results, a receiver operating characteristic curve was constructed using the final elasticity measurements as a predictor with an accompanying 95% confidence interval for the AUC.

Similarly, for each grade of steatosis, a receiver operating characteristic curve, and an accompanying 95% confidence interval for the AUC, was constructed using the attenuation or VDFF measures as the predictor. The AUC’s for Velacur and FibroScan were calculated and compared.

Analysis was completed using MATLAB (Mathworks, Natick, MA, USA), version 2022b. 

## 3. Results

### 3.1. Patient Characteristics

A total of 78 patients were screened and enrolled in this study. Although scheduled at the time of enrollment, 5% (4/78) of patients did not complete the biopsy or had a biopsy more than 6 months from the Velacur scan. The original window of 3 months was expanded to 6 months to capture additional patients in this study without affecting the overall results (see Discussion). Of the patients enrolled, 5% (4/78) had invalid Velacur scans.

**Figure 1:**
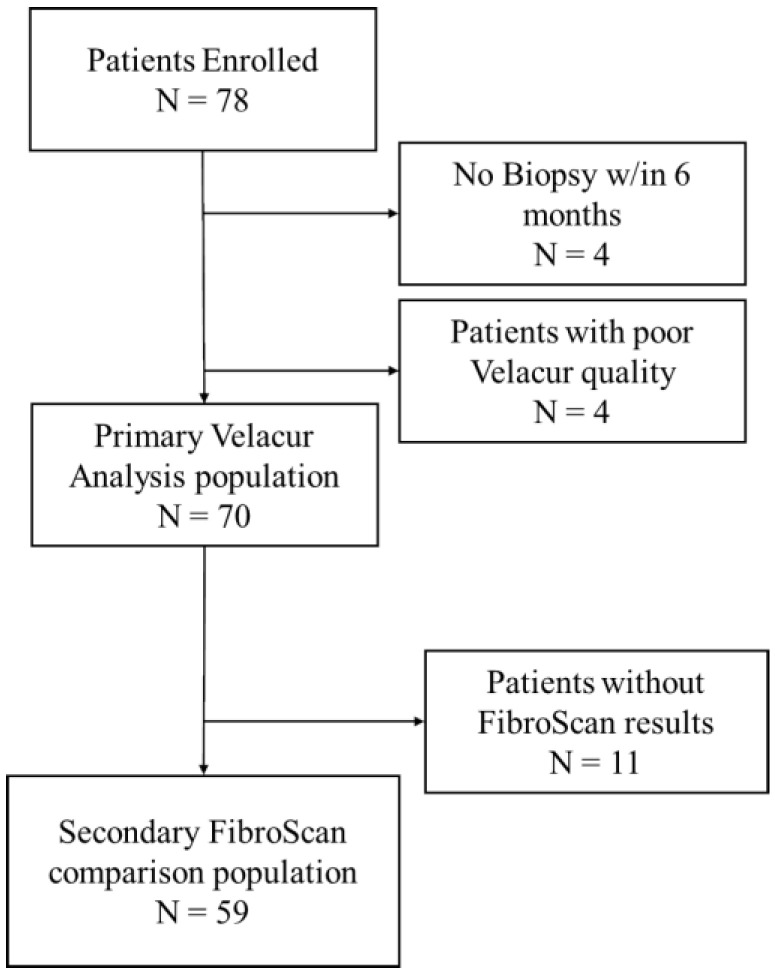
Patient flow diagram showing the total number of patients in each analysis population and the reasons for exclusion.

Table 1 outlines the patient characteristics of all patient populations, and Figure 1 shows the number of patients included and excluded for each population. The Velacur analysis population included those patients with both a valid biopsy and a valid Velacur result. The average age of the patients in this population was 53.3 ± 13.1 years, with a mean BMI of 35.0 ± 6.24 kg/m^2^. The mean time between the Velacur scans and biopsy was 42.7 ± 41.4 days. The mean Velacur stiffness was 6.48 ± 1.4 kPa, and the mean VDFF was 14.4 ± 5.1%. Five patients (7%) were missing part of their bloodwork, and the Fib-4 score was not calculated. These patients were deemed to be random, and missing values were ignored in the overall results presented in Table 1.

As part of this study, some of the patients with FibroScan results were also included in the analysis of the secondary objective. Of the 78 enrolled patients, 76% (59/78) had a FibroScan result within 6 months from their scan. Table 1 outlines the patient characteristics of this population as well.

### 3.2. Discriminatory Ability for Fibrosis

**Figure 2:**
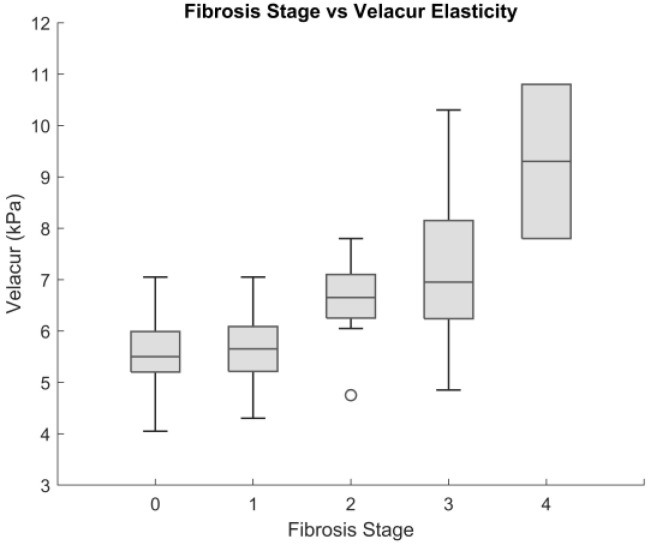
Velacur results of patients with each stage of fibrosis as defined by biopsy.

A total of 11, 19, 13, 25, and 2 patients in the Velacur analysis population were categorized as F0 to F4, respectively, according to the biopsy reports. All pathologists used the NASH CRN scoring system, as described by Kleiner et al. and Brunt et al. [26,27]. Patients with stage 1a, 1b, or 1c were included into F1; six patients had this sub classification. Only two patients were reported at F4, or cirrhosis. The AUC for each stage (≥F1, ≥F2, ≥F3, and F4) was 0.75, 0.86, 0.79, and 0.94, respectively. Figure 2 shows the box–whisker plot for the Velacur results of patients with each stage of fibrosis. Table 2 provides an overview of the number of patients and the AUC results.

### 3.3. Discriminatory Ability for Steatosis

**Figure 3:**
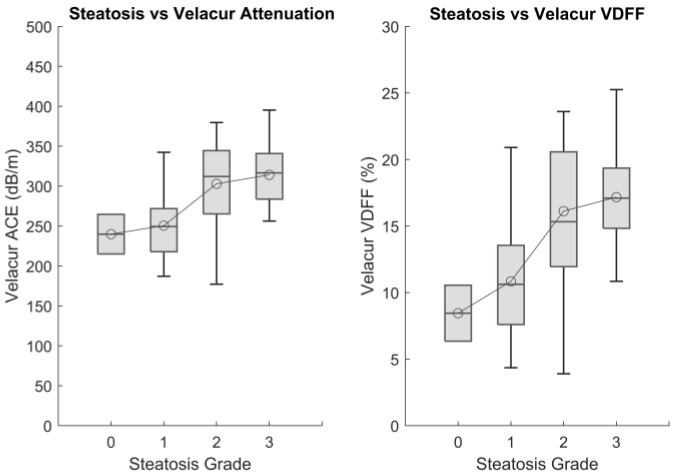
Velacur ACE (**Left**) and VDFF (**right**) results for patients with each steatosis grade as defined by biopsy.

A total of 2, 24, 20, and 24 patients in the Velacur analysis population were categorized as S0 to S3, respectively. As all patients had MASLD or MASH at the time of biopsy, very few patients were found to be in the S0 category. The AUC for discriminatory ability for each grade (≥S1, ≥S2, and S3) for Velacur ACE and VDFF are outlined in Table 3. Figure 3 shows the box–whisker plot for Velacur results of patients with each grade of steatosis.

### 3.4. Comparison to FibroScan

A subset of patients (n = 59) had FibroScan results that were within 6 months of the biopsy and were included in the analysis. The AUC for fibrosis stages and steatosis grades for both Velacur and FibroScan were calculated.

Table 4 outlines the results for these patients. Delong’s test was used to determine if the AUC results are significantly different.

These results for FibroScan are in a smaller population than in other studies so a comparison of the Velacur results to the previous literature for CAP results was also undertaken. When comparing the Velecur results to previous results for FibroScan from the literature [28,29] on larger populations, Velacur performs favorably in comparison. The AUC detection of any fibrosis was 0.82 (0.76–0.88) [28] and 0.67 (0.56–0.78) [29]. For significant fibrosis, the results were 0.86 (0.82–0.91) [28] and 0.86 (0.77–0.95) [29], and for advanced fibrosis, the results were 0.84 (0.78–0.90) [28] and 0.80 (0.67–0.93) [29].

## 4. Discussion

The results of this validation study are in line with previously published results [22]. Although the previous results with Velacur were using MRE and MRI-PDFF as the reference standard, the results presented here show that Velacur can be used as a non-invasive surrogate for patient assessment when using biopsy as the gold standard as well. Previously, when using MRI elasticity as the standard for fibrosis assessment [28], Velacur results reported AUC’s of 0.83, 0.88, 0.95, and 0.97 for the detection of ≥F1, ≥F2, ≥F3, and F4, respectively. These previous results are similar to those found in this study, with an understanding of the limitations of the biopsy reading and the conversion of MRE stiffness values to categorical fibrosis scoring using cutoffs defined in other studies [28].

Although biopsy is currently used within a clinical trial context and the only definitive way to diagnose MASH, it remains suboptimal [30]. Low inter- and intra-rater scores have led to complicated methods for creating consensus, involving multiple pathologists and requiring consensus meetings regarding patients with inconsistent readings. One limitation of this study was that the majority of biopsies were read by a single pathologist at a single time point, rather than using a consensus strategy [20]. This represents a scenario that is more realistic to general clinical practice but may introduce additional variability and uncertainty around the scores for fibrosis and steatosis.

As patients were not randomly selected to undergo biopsy, there is a selection bias among these patients. Patients were undergoing biopsy as part of clinical care or assessment for pharmaceutical clinical trials. Of the patients included in this study, 6 were not part of a pharmaceutical clinical trial, and at least 17 patients had failed screening for a trial and did not start on any treatment. Of the others, the biopsy was performed before, during, or at the end of the trial. Due to blinding, it is unknown if the patients were part of the treatment or placebo arms of the study. To assess the possible influence treatment could have had on the results presented here, an analysis of the change in accuracy depending on the time between biopsy and Velacur scans was undertaken. The AUROC for significant and advanced fibrosis, as well as for significant steatosis, was examined using different time limits, from 6 months to 1 month. There were no significant differences in the results as the time limit shrank, with variations of less than 0.02 in the AUROC.

The number of patients in each category was not evenly distributed, with higher numbers of patients in the F2 and F3 categories and in the higher grades of steatosis. These are often the patients who require additional information about the disease status from biopsy or may qualify for clinical studies. The low number of cirrhotic patients, and patients labeled as ‘S0’, limit the interpretation of the results in this context, but these are also the patients least likely to undergo biopsy in any clinical situation.

Another limitation of this study was that all patients enrolled in this study were from a single site, with a high Hispanic population. Although this may be considered a strength of the study, as MASH prevalence is very high in Hispanics and they are a target for MASH treatment and diagnosis [31], we will continue to validate Velacur performance in more diverse populations.

Some patients were removed from the analysis population due to invalid Velacur scans. The invalid scans were marked by the objective shear wave quality metric and are typically caused by improper ultrasound probe placement, such that not enough of the liver is visible in the ultrasound image. All operators were trained immediately prior to starting this study. The patients with invalid scans were scanned within the first 15 patients enrolled and were likely invalid due to the learning curve of the operator. Of the last half of patients enrolled, none were removed as invalid. The training process and learning curves for Velacur continue to be assessed in future work.

## 5. Conclusions

Although non-invasive methods are becoming the standard of care, biopsy remains the only definitive method of MASH diagnosis. Thus, it remains an important benchmark for validation of new non-invasive biomarkers.

This validation of Velacur elasticity, attenuation, and VDFF measures against histological results proves that Velacur is a valid method to assess patients with Metabolic Dysfunction-Associated Steatotic Liver Disease and Metabolic Dysfunction-Associated Steatohepatitis.

## Figures and Tables

**Table 1:** Patient characteristics for each analysis population.

Characteristics	All Subjects with Biopsy Results	Subjects in Velacur Analysis	Subjects in FibroScan Comparison
Total Enrolled Subjects	78	78	78
Total Analyzed Subjects	74	70	59
Age (years, mean ± std)	54.0 ± 13.1	53.3 ± 13.1	53.3 ± 13.7
Gender (% Female)	48.6	45.7	49.2
BMI (kg/m^2^, mean ± std)	35.1 ± 6.23	35.0 ± 6.24	35.3 ± 6.35
Diabetes (% yes)	43.2	41.4	39
Hypertension (% yes)	58.1	55.7	55.9
Fib-4 score ^1^ (mean ± std)	1.22 ± 0.6	1.20 ± 0.6	1.23 ± 0.6
Race ^2^ (% Caucasian)	28.4	28.6	30.5
Race ^2^ (% Hispanic)	55.4	54.3	54.2
Race ^2^ (% Asian and other)	13.5	14.3	11.9
Velacur Elasticity (kPa, mean ± std)		6.48 ± 1.41	6.55 ± 1.49
Velacur ACE (dB/m, mean ± std)		287 ± 51.2	287 ± 50.1
Velacur VDFF (%, mean ± std)		14.4 ± 5.14	14.5 ± 5.03
Time from Biopsy to Velacur (days, mean ± std)		42.7 ± 41.4	38.3 ± 32
FibroScan Elasticity (kPa, mean ± std)			13.6 ± 10.6
FibroScan CAP (dB/m, mean ± std)			333 ± 43.6
M probe (%)			32.2
Time from Biopsy to FibroScan (days, mean ± std)			48.7 ± 34.4
Patients with Fibrosis Stage ^3^ (N)	11, 20, 14, 27, 2	11, 19, 13, 25, 2	9, 17, 11, 20, 2
Patients with Steatosis Grade (N)	3, 26, 21, 24	2, 24, 20, 24	0, 23, 16, 20

^1^ Calculated from most recent bloodwork with all components. ^2^ Patients were allowed to indicate all which applied. ^3^ Patients with stage 1a, 1b, and 1c are included as stage F1.

**Table 2:** Patient numbers and Velacur AUC results for discrimination of each fibrosis stage.

Fibrosis Stage	N	Discrimination	AUC [95% CI]
F0	11		
F1	19	F0 vs. F1-F4	0.753 [0.583, 0.877]
F2	13	F0-F1 vs. F2-F4	0.861 [0.765, 0.934]
F3	25	F0-F2 vs. F3-F4	0.791 [0.656, 0.884]
F4	2	F0-F3 vs. F4	0.941 [0.806, 1.000]

**Table 3:** Patient numbers and Velacur AUC results for discrimination of each steatosis grade.

Steatosis Grade	N	Discrimination	ACE AUC [95% CI]	VDFF AUC [95% CI]
S0	2			
S1	24	S0 vs. S1-S3	0.765 [0.522, 0.928]	0.868 [0.725, 0.956]
S2	20	S0-S1 vs. S2-S3	0.848 [0.740, 0.929]	0.846 [0.721, 0.920]
S3	24	S0-S2 vs. S3	0.743 [0.627, 0.841]	0.740 [0.606, 0.838]

**Table 4:** The AUROC comparisons for patients with both FibroScan and Velacur results.

	Patients (N)	Velacur AUROC [95% CI]	FibroScan AUROC [95% CI]	*p*-Value
**Velacur Elasticity and FibroScan Elasticity**	**59 total**			
Detection of significant fibrosis (≥F2)	26 vs. 33	0.89 [0.78, 0.95]	0.76 [0.59, 0.86]	0.072
Detection of advanced fibrosis (≥F3)	37 vs. 22	0.83 [0.68, 0.92]	0.75 [0.58, 0.86]	0.276
**Velacur VDFF and FibroScan CAP**	**59 total**			
Detection of moderate steatosis (≥S2)	23 vs. 36	0.85 [0.72, 0.94]	0.50 [0.35, 0.66]	<0.001
Detection of severe steatosis (≥S3)	39 vs. 20	0.74 [0.60, 0.86]	0.57 [0.39, 0.73]	0.061

## Data Availability

The raw data supporting the conclusions of this article will be made available by the authors on request.
